# Distribution dataset of chondrichthyan species occurring in French metropolitan waters and across the North-East Atlantic

**DOI:** 10.3897/BDJ.14.e191625

**Published:** 2026-09-08

**Authors:** Amaëlle Bisch, Pauline Stephan, Sophie Elliott, Thomas Barreau, Caroline Bousquet, Laurent Colombet, Ghislain Dorémus, Michel Marengo, Matthieu Lapinski, Alexandra Rohr, François Sichel, Eric Stephan, Anthony Acou

**Affiliations:** 1 UAR OFB-MNHN-CNRS-IRD PatriNat, Station marine de Dinard, Dinard, France UAR OFB-MNHN-CNRS-IRD PatriNat, Station marine de Dinard Dinard France; 2 Durrell Institute of Conservation and Ecology (DICE), School of Natural Science, University of Kent, Canterbury, CT2 7NR, United Kingdom Durrell Institute of Conservation and Ecology (DICE), School of Natural Science, University of Kent Canterbury, CT2 7NR United Kingdom; 3 MNHN, Station marine de Dinard, Dinard, France MNHN, Station marine de Dinard Dinard France; 4 Université de Corse Pasquale Paoli, Corte, France Université de Corse Pasquale Paoli Corte France; 5 Association Les Amis de BioObs, Mèze, France Association Les Amis de BioObs Mèze France; 6 Observatoire Pelagis – La Rochelle Université / CNRS, La Rochelle, France Observatoire Pelagis – La Rochelle Université / CNRS La Rochelle France; 7 Station de Recherches Sous-marines et Océanographiques de Calvi (STARESO), Calvi, France Station de Recherches Sous-marines et Océanographiques de Calvi (STARESO) Calvi France; 8 Association Ailerons, Montpellier, France Association Ailerons Montpellier France; 9 Association pour l’étude et la conservation des sélaciens (APECS), Brest, France Association pour l’étude et la conservation des sélaciens (APECS) Brest France; 10 ABTE UR4651, Université de Caen-Normandie, Caen, France ABTE UR4651, Université de Caen-Normandie Caen France

**Keywords:** biodiversity, chimaera, citizen science, fisheries-dependent data, Marine Strategy Framework Directive, presence-absence, ray, scientific surveys, shark, skate

## Abstract

**Background:**

Chondrichthyans (chimaeras, sharks, skates and rays) are the most threatened vertebrate group in the marine environment. Currently, 106 species (7 chimaeras, 40 skates and rays, 59 sharks) of chondrichthyans are known to occur regularly or occasionally in metropolitan French waters. However, the distribution of these species remains poorly documented due to a lack of data. In this context, a comprehensive dataset was compiled to support the recently published *Atlas des Chondrichtyens de France métropolitaine* (Chondrichthyan Distribution Atlas within North-East Atlantic and French Mediterranean waters), aiming to provide an updated and standardised overview of their occurrence in French metropolitan and adjacent marine waters.

**New information:**

This dataset compiles 177,042 events (e.g. fishing operations, transects, sightings) collected between 2003 and 2021 across the North-East Atlantic (Greater North Sea, Celtic Seas, Bay of Biscay and Iberian Coast) and Western Mediterranean Sea. These events comprise 169,519 occurrence records representing 86 chondrichthyan species (7 chimaeras, 35 skates and rays, 44 sharks). The dataset includes fisheries-dependent data, scientific survey data and citizen-science observations. Each record was carefully checked for taxonomic accuracy, geographical plausibility, depth, and biological (i.e. individual length) compliance with existing literature. French experts further reviewed the reliability of occurrences for the species within their taxonomic area of expertise. The dataset is publicly available through the Global Biodiversity Information Facility (GBIF) and can be found under: https://doi.org/10.15468/xq94ag.

## Introduction

Chondrichthyans are key components of marine ecosystems, playing important ecological roles, such as top-down regulatory effects on prey population (Baum and Worm 2009, Ferretti et al. 2010). They are the most threatened vertebrate group in the marine environment and the second most threatened vertebrate group globally, after amphibians (Dulvy et al. 2021). However, due to their low fecundity, slow growth and late sexual maturity, many chondrichthyan species are under significant pressure from fishing, habitat degradation and climate change (Frisk et al. 2001, Walls and Dulvy 2021, Coulon et al. 2025). France hosts a rich and diverse chondrichthyan fauna, representative of both the North-East Atlantic and Western Mediterranean Sea communities. While in Europe, 32% of chondrichthyan species are listed as Threatened on the IUCN Red List (Nieto et al. 2015), more than half of the species known to occur in French metropolitan waters were classified in 2013 as ‘Data Deficient’ on the French IUCN Red List (UICN France and MNHN 2013), highlighting major gaps in knowledge regarding their distribution and population status.

These knowledge gaps reflect the absence of comprehensive, long-term monitoring programmes specifically dedicated to chondrichthyans across the North-East Atlantic and Western Mediterranean Sea. Available information largely derives from fisheries-independent scientific surveys (e.g. scientific bottom trawl surveys), fisheries-dependent observer programmes and opportunistic citizen-science records, which differ in sampling design, effort, spatial coverage and taxonomic accuracy. This heterogeneity makes large-scale synthesis and temporal trend assessment particularly challenging (Boakes et al. 2010, Isaac and Pocock 2015). This lack of harmonised information limits the ability to assess the conservation status of these species and to implement effective management measures (Dulvy et al. 2014, Cazalis et al. 2022).

The Marine Strategy Framework Directive (MSFD; 2008/56/EC) recommends that Member States take concerted measures to consider all components of the marine ecosystem, to maintain or restore the good functioning of the marine ecosystem and achieving good environmental status (GES) of the marine environment. Under the MSFD’s Descriptor 1, which focuses on maintaining biodiversity, France initiated a dedicated action to consolidate all available information on national occurrences of chondrichthyans. This initiative for French metropolitan waters (extended to adjacent waters due to the mobility of species), sought to provide a coherent, quality-controlled dataset serving as a basis for assessing species distribution, occurrence frequency and potential hotspots of increased conservation interest.

The dataset presented here compiles, harmonises and assesses the reliability of occurrence records of chondrichthyan species from events (e.g. fishing operation, transect, sighting) in the Greater North Sea (including the Kattegat, the Skagerrak and English Channel), Celtic Seas, Bay of Biscay and the Iberian Coast and Western Mediterranean Sea, between 2003 and 2021. This unified dataset was the foundation for the *Atlas des Chondrichtyens de France métropolitaine* (Bisch et al. 2024), providing the first standardised and spatially comprehensive synthesis of these species’ occurrences. Beyond its role in the Atlas, this dataset offers a reusable and open-access resource to support ongoing and future research, conservation planning and policy implementation related to marine biodiversity in the North-East Atlantic and Western Mediterranean Sea.

## General description

### Purpose

The purpose of this dataset is to compile, clean and standardise occurrence data between 2003 and 2021 for chondrichthyans known to occur in French metropolitan marine waters including occasional records from estuarine environments. Given the mobile nature of these species, available data at a North-East Atlantic and Western Mediterranean Sea scale are also considered. It serves as a basis for scientific analysis, conservation planning and reporting under national and European regulations (i.e. the MSFD).

### Additional information

The dataset consolidates occurrence information from three complementary sources: (i) fisheries-dependent data (i.e. the French professional fisheries observer programme); (ii) scientific surveys, including bottom trawl and acoustic surveys, deep-sea canyon exploration cruises, aerial and vessel-based observation surveys and (iii) citizen-science initiatives (e.g. diver observations, recreational sightings and public reporting schemes). All occurrence records underwent a reliability assessment. For fisheries-dependent data (excluding Corsican data) and scientific survey data, this included taxonomic verification and checks of spatial plausibility, depth consistency and biological realism (i.e. reported individual lengths). In addition, French taxonomic experts assessed the reliability of occurrences for the species falling within their area of expertise. For fisheries-dependent data and citizen-science initiatives in Corsica, data providers undertook their own quality control procedures prior to provision.

## Sampling methods

### Study extent

The dataset comprises fishing operations and observations collected from various sources, including the French professional fisheries observer programme (ObsMer), scientific surveys and citizen-science programmes (Table 1). All data compiled from these sources are temporally aligned with the ObsMer programme and the initiation of the French chondrichthyans distribution Atlas project. Each entry in the dataset corresponds to a single, uniquely identified record. This record represents either a sampling event (for presence/absence sources) or a single occurrence record (for presence-only data), depending on the origin of the data (Table 1). In all cases, a presence (or occurrence) represents a point of contact with the species, whether through a fishing operation or an observation, regardless of the number of individuals recorded at that location. The spatial resolution of each dataset varies depending on the sampling strategy and/or data anonymisation requirements. When the exact coordinates were recorded, the occurrences are projected directly (i.e. citizen-science data, as well as aerial and boat surveys conducted by Pelagis, a French organisation dedicated to marine megafauna monitoring). For fishing operations, when GPS coordinates for the shooting and the hauling events were available, the centroid of both coordinates was used (i.e. scientific bottom trawl surveys, acoustic trawl surveys). For the MedSeaCan and CorSeaCan explorations, global GPS coordinates of the canyon or rock head were used instead of exact dive locations. Due to data sensitivity, ObsMer fishing operations are reported at the International Council for the Exploration of the Sea (ICES) statistical subrectangle (0.16 degree of latitude by 0.33 degree of longitude) rather than precise GPS coordinates. Any operations or observations that lacked GPS coordinates or overlapped land areas were removed from the dataset.

### Sampling description

Each data source compiled in this dataset has a distinct sampling strategy. A brief description of the sampling procedure for each source is provided below. For more details, please refer to the links included in each description.

- **French professional fisheries observer programmes**:

ObsMer data collection is carried out by observers on board professional fishing vessels of various sizes, following a stratified sampling approach, based on geography, season (quarterly), vessel size class and fleets. Vessels are randomly selected and subject to the approval of the fishing skipper. The spatio-temporal sampling coverage by métier (defined by fishing gear, target species and fishing zone) is designed to be representative of the activity of the French fishing fleets. Observers sample three categories of catches: landings, discards and bycatches. Depending on the category, they identify to species level, weigh, count and/or measure individuals from a representative sample of the fishing operation. The manual for observers on board commercial fishing vessels is available at the following link: https://archimer.ifremer.fr/doc/00664/77630/.

The fishing operations included in this dataset primarily cover the Exclusive Economic Zones (EEZs) of France and Great Britain (including Jersey and Guernsey), as well as Spain, Belgium, Ireland and Norway. Some localised fishing operations on the high seas were also sampled. It is worth noting that small-scale fishing monitoring in Corsica under ObsMer was conducted by the "Station de Recherches Sous-Marines et Océanographiques de Calvi" (STARESO) between 2012 and 2014. For 2009-2011 and 2015, STARESO carried out the monitoring of small-scale fisheries independently of the ObsMer programme. Since the monitoring protocol used by STARESO was comparable to that of the ObsMer programme, these records were grouped into a single dataset. Additional programmes monitoring small-scale fishing in Corsica were carried out after 2015, but are not included in this dataset.

- **Scientific bottom trawl surveys**:

Scientific bottom trawl surveys follow standardised protocols for gear, fishing duration and operation speed and they are conducted in the same areas at the same time each year. All surveys use bottom trawls, primarily detecting benthic and demersal species. Most do not sample beyond 500 metres depth. In the North-East Atlantic, scientific bottom trawl data are centralised by ICES through the Database on Trawl Surveys (DATRAS; https://www.ices.dk/data/data-portals/pages/datras.aspx). In the Western Mediterranean Sea, the MEDITS survey (MEDIterranean Trawl Survey; https://sextant.ifremer.fr/record/a5d075c0-29a1-43ab-a810-18a07c04f5d3/) is deployed. The operations included in this dataset cover the following MSFD areas: the Greater North Sea (including the Kattegat, the Skagerrak and the English Channel), Celtic Seas, Bay of Biscay and the Iberian Coast and the Western Mediterranean Sea (i.e. the Gulf of Lion and eastern Corsica).

- **Acoustic trawl surveys**:

Acoustic trawl surveys targeting commercial pelagic fish (e.g. anchovy, mackerel, sardines) follow standardised protocols for gear, fishing duration and operation speed and are conducted in the same areas at the same time each year. Pelagic fish species are detected using echosounders and species identification (trawl verification) is carried out through accompanying pelagic trawl hauls (Doray et al. 2021). During these trawling operations, elasmobranchs may occur as bycatch. Acoustic trawl survey data are held by ICES (https://www.ices.dk/data/data-portals/Pages/acoustic.aspx). The operations included in this dataset cover the Greater North Sea and the Bay of Biscay.

- **MedSeaCan/CorSeaCan**:

MedSeaCan and CorSeaCan campaigns exploring the Mediterranean canyons consisted of a standardised sampling effort of approximately ten days per expedition, across 13 selected areas each encompassing multiple canyons and/or deep-sea rocky habitats (Goujard and Fourt 2014). The explorations were conducted using a ROV or a two-seater submarine, during which photos, videos and biological samples were collected. Survey depths ranged from 90 to 700 metres, covering the Gulf of Lion and Corsica.

- **Pelagis aerial surveys**:

Pelagis aerial surveys collected surface observations at an altitude of 180 metres and a speed of 167 km/h (90 knots). Two observers scan the sea surface with the naked eye, recording seabirds and macro-waste within a 200-metre strip on either side of the aircraft along the transects. Marine mammals, turtles and large fish observed (e.g. pelagic elasmobranchs) at the surface are documented without any distance limitation. Environmental factors, such as weather conditions and turbidity, which can have significant impact on species detection, are also recorded. These aerial surveys are primarily conducted within the French EEZ in Atlantic and Mediterranean waters. The full aerial monitoring protocol is available at the following link: https://www.observatoire-pelagis.cnrs.fr/wp-content/uploads/2021/08/GuideMethodoAerien2020.pdf.

- **Pelagis boat surveys**:

Pelagis marine megafauna monitoring programme (https://www.observatoire-pelagis.cnrs.fr/suivis-en-mer/suivis-par-bateau/) is conducted aboard vessels of the French oceanographic fleet during various scientific surveys (Table 1), including scientific bottom trawl surveys (CGFS, EVHOE), acoustic trawl surveys (PELGAS, PELMED) and the maintenance of Mediterranean deep-sea anchorages (MOOSE-GE). For each surveyed area, one or two dedicated observers record observation conditions, document the presence of megafauna (e.g. cetaceans, seabirds, turtles, large pelagic fish) and log objects encountered such as macro-waste and ships. These observations primarily cover the French EEZ in both Atlantic and Mediterranean waters.

- **Citizen science**:

Citizen-science observation data come from the ELASMED observatory, managed by the Ailerons association (https://www.asso-ailerons.fr/), as well as BioObs (https://bioobs.fr/blog/) and the national basking shark public sightings recording scheme run by the APECS association (https://asso-apecs.org/actions/programme-national-de-recensement-des-requins-pelerins/). Unlike structured scientific surveys, citizen-science observations do not follow standardised sampling protocols. These data include opportunistic observations reported by divers, recreational fishers and other sea users covering the Greater North Sea (including the Kattegat, the Skagerrak and the English Channel), Celtic Seas, Bay of Biscay and the Iberian Coast, as well as the Western Mediterranean Sea for data from BioObs and APECS. ELASMED data are limited to the Western Mediterranean Sea.

### Quality control

All occurrences included in this dataset underwent a reliability assessment. For citizen-science data (i.e. ELASMED, BioObs, APECS) and the professional fisheries observer programme in Corsica (ObsMer operated by STARESO), quality control was performed by the organisations managing these data to ensure that only valid occurrences were retained. Validation procedures varied amongst data providers, but generally included expert review and, where available, supporting material such as photographs. For example, BioObs records undergo a double validation process that combines checks for geographic consistency, feasibility of the observation during diving activities and photographic verification when available. Similarly, APECS records are validated following a standardised protocol (Eloire et al. 2022) and only observations meeting defined reliability levels (i.e. 1a, 1b, 2) were included in the dataset. For all other data sources (i.e. ObsMer data from the North-East Atlantic and the Gulf of Lion, scientific bottom trawl and acoustic trawl surveys, MedSeaCan/CorSeaCan campaigns, Pelagis aerial and boat surveys), each species occurrence was individually assessed for reliability. This process involved verifying that key attributes from the initial datasets, such as location, individual length and capture depth, were consistent with expected ranges extracted from literature.

Between two and five references were used for each species to assess the geographical plausibility of the occurrences. The complete set of references used for this purpose was: Last et al. (2016), Scientific, Technical and Economic Committee for Fisheries (STECF) (2017), ICES (2020), Ebert and Dando (2022), IUCN (2022) and ICES stock information sheets. Within this set, references were considered relevant for a species only if they documented that given species. An occurrence was classified as geographically plausible when more than 50% of the relevant references confirmed the presence of the species within the ICES division where it was recorded.

To validate the length and depth information for all recorded species, Weigmann (2016) was used as a reference. For individual lengths, an occurrence was considered consistent when the recorded length was less than or equal to the maximum reference length. When several individuals were measured within the same occurrence, more than 50% of the measurements had to fall within expected ranges for the occurrence to be considered consistent in length. An occurrence was considered consistent in depth when the capture depth value fell within the reference depth range or did not exceed the maximum reference depth. For deep-water species (i.e. bathydemersal and bathypelagic), both minimum and maximum depth thresholds were assessed. For all other species, only the maximum depth threshold was considered.

The thresholds used were deliberately defined as fixed reference values to ensure a consistent and reproducible validation framework across all species. Species specific deviations from known biological or ecological ranges can occur, particularly in the context of range shifts or rare observations. For consistency, occurrence reliability was assessed by combining multiple lines of evidence (i.e. geographical plausibility, depth consistency and biological realism). In cases where records were considered ambiguous or borderline, expert judgement was systematically used to support the final assessment. Based on the reliability assessment and expert judgement, four levels of certainty were assigned to each occurrence (Table 2).

## Geographic coverage

### Description

**Figure 1.**
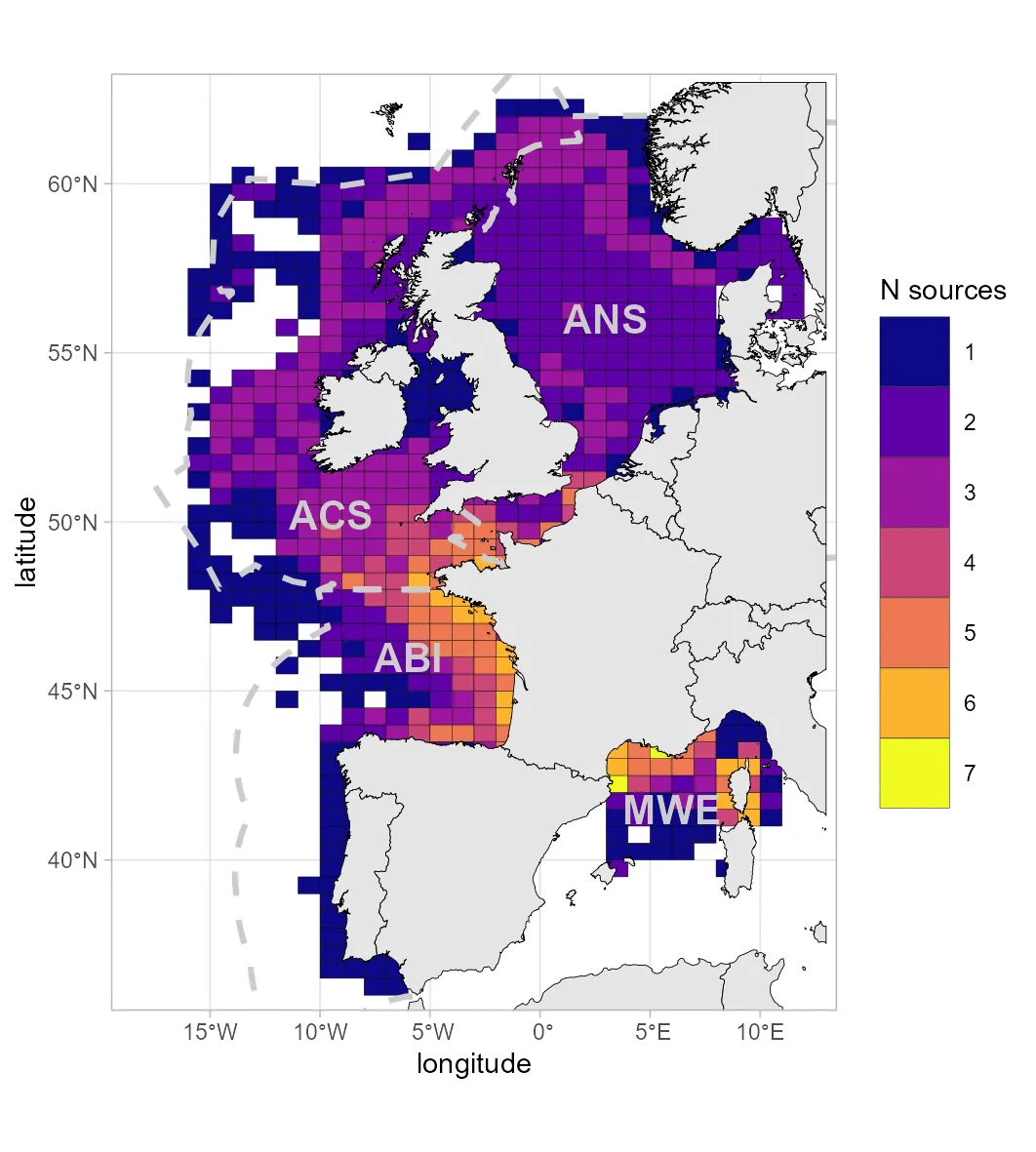
Spatial coverage and number of data sources (from one to seven maximum) available at the ICES statistical rectangle level. Grey dashed lines delineate MSFD regions. ANS: Greater North Sea; ACS: Celtic Seas; ABI: Bay of Biscay and Iberian coast; MWE: Western Mediterranean Sea.

The dataset covers the four marine sub-regions defined under the MSFD: Greater North Sea (including Kattegat, the Skagerrak and English Channel), Celtic Seas, Bay of Biscay and the Iberian Coast and Western Mediterranean Sea (Fig. 1), where sufficient data existed. Where data existed just outside the MSFD region, these were also included.

### Coordinates

36.00 and 62.33 Latitude; -15.93 and 12.86 Longitude.

## Taxonomic coverage

### Description

The dataset includes 86 chondrichthyan species (7 Holocephali, 79 Elasmobranchii) known to occur in French marine metropolitan waters (Table 3). This number is lower than the total number of species reported for these waters (106) because some species were not recorded in the compiled data sources during the study period (2003–2021). The latter most likely reflects their rarity, limited detectability or absence from the surveyed habitats. All the scientific names of the species comply with the TAXREF v.18 reference database (Gargominy 2025) which is the national taxonomic reference database for the fauna, flora and fungi of metropolitan France and its overseas territories, developed and published by the Museum national d'Histoire naturelle (MNHN) as part of the implementation of the National Natural Heritage Inventory Information System (SINP).

## Temporal coverage

**Data range:** 2003-1-08 – 2021-12-21.

## Usage licence

### Usage licence

Creative Commons Public Domain Waiver (CC-Zero)

## Data resources

### Data package title

French chondrichthyans distribution dataset in North-Eastern Atlantic and Western Mediterranean waters (2003–2021)

### Resource link

https://doi.org/10.15468/xq94ag

### Alternative identifiers

https://ipt.gbif.fr/resource?r=french_elasmo_dataset, https://www.gbif.org/dataset/db8b1693-ed9e-4bb2-a737-62dcd8d7839d

### Number of data sets

2

### Data set 1.

#### Data set name

event

#### Description

This dataset compiles all ‘events’ (e.g. fishing operations, observations) and their characteristics (e.g. gear/method, date, location). This dataset is linked to the second dataset 'occurrence' by the eventID. When an event is not linked to the 'occurrence' dataset, it indicates that there was no capture or observation of the species of interest during that event.

**Data set 1.** 

Column label	Column description
datasetID	DOI or unique identifier of the dataset, when available.
datasetName	The name identifying the dataset from which the event was derived.
ownerInstitutionCode	The name or acronym used by the institution that owns the dataset.
dataGeneralizations	Measures taken to make shared data less specific in the case of sensitive data or measures taken to simplify raw information in the case of non-sensitive data.
eventID	Unique identifier for the event (e.g. fishing operation, transect, sighting etc.). Used as a key to join the two datasets.
eventDate	Date of the event. Can be in Year-Month, Year-Month-Day format or a range depending on the information available in the data source.
samplingProtocol	Fishing gear or observation method used during the event.
locationID	Subdivision of the ICES statistical rectangle where the event took place.
higherGeography	MSFD area where the event took place.
waterBody	Waterbody where the event took place.
countryCode	Country code of the EEZ where the event took place according to ISO 3166-1-alpha-2 country code.
decimalLatitude	The geographic latitude of the event (in decimal degrees, using the spatial reference system given in geodeticDatum). When this field is not filled in, see locationID, footprintWKT, footprintSRS and dataGeneralizations.
decimalLongitude	The geographic longitude of the event (in decimal degrees, using the spatial reference system given in geodeticDatum). When this field is not filled in, see locationID, footprintWKT, footprintSRS and dataGeneralizations.
geodeticDatum	The spatial reference system (SRS) upon which the geographic coordinates given in decimalLatitude and decimalLongitude are based.
footprintWKT	A Well-Known Text (WKT) representation of the shape (footprint, geometry) that defines the locationID.
footprintSRS	The spatial reference system (SRS) upon which the geometry given in footprintWKT is based.

### Data set 2.

#### Data set name

occurrence

#### Description

This dataset compiles all occurrences of species of interest. An occurrence is defined here as "species encountered" without considering the number of individuals encountered.

**Data set 2.** 

Column label	Column description
basisOfRecord	The specific nature of the data record.
occurrenceID	Unique identifier for the occurrence.
eventID	Unique identifier for the event (e.g. fishing operation, transect, sighting etc.). Used as a key to join the two datasets.
identificationVerificationStatus	Level of certainty of the occurrence. For more details, see the “quality control” section.
scientificName	Scientific name of the species. All scientific names of species comply with the TAXREF v.18 reference database.
kingdom	Species kingdom.
phylum	Species phylum.
class	Species class.
order	Species order.
family	Species family.
scientificNameAuthorship	The authorship information for the scientificName.

## Figures and Tables

**Table 1.** Summary of the sources in the database. P/A: presence/absence; P-only: presence-only; N_events_: number of events; N_occ_: number of occurrences.

**Source type**	**Data type**	**Data source**	**DOI or identifier (when available)**	**Data owner**	**N_events_**	**N_occ_**
Fisheries- dependent	P/A	French professional fisheries observer programmes (ObsMer and STARESO monitoring)	ObsMer: https://doi.org/10.12770/24538369-ea5a-48d7-a89b-0c9530247ed2	Direction générale des affaires maritimes, de la pêche et de l'aquaculture (DGAMPA) Corsican data only: Station de Recherches Sous-Marines et Océanographiques de Calvi (STARESO) data owner for 2009–2011 and 2015.	109,750	87,491
Scientific survey	P/A	Scientific bottom trawl surveys	North-East Atlantic: https://doi.org/10.17895/ices.pub.23634315 Western Mediterranean Sea: https://doi.org/10.18142/7	North-East Atlantic: International Council for the Exploration of the Sea (ICES) Western Mediterranean Sea: IFREMER	58,753	76,452
Scientific survey	P/A	Acoustic trawl surveys	https://gis.ices.dk/geonetwork/srv/metadata/8f4651a7-904c-4bac-95dc-414312f24527	ICES	3,138	175
Scientific survey	P/A, but presented in this dataset as P-only	MedSeaCan and CorSeaCan		Agence des aires marines protégées - Office français de la biodiversité (OFB)	94	94
Scientific survey	P/A, but presented in this dataset as P-only	Pelagis aerial surveys	https://doi.org/10.82144/436ef8df	Observatoire Pelagis (La Rochelle Université - CNRS)	1,703	1,703
Scientific survey	P/A, but presented in this dataset as P-only	Pelagis boat surveys	https://doi.org/10.82144/c7d01c61 Data collected from following surveys: - Channel Groundfish Survey (CGFS): https://doi.org/10.18142/11 - Évaluation des ressources Halieutiques de l'Ouest de l'Europe (EVHOE): https://doi.org/10.17600/18002363 - Pélagiques Gascogne (PELGAS): https://doi.org/10.17882/53389 - Pélagiques Méditerranée (PELMED): https://doi.org/10.18142/19 - Mediterranean Ocean Observing System for the Environment (MOOSE-GE): https://doi.org/10.18142/235	Observatoire Pelagis (La Rochelle Université - CNRS)	116	116
Citizen science	P-only	ELASMED		Ailerons association	1,160	1,160
Citizen science	P-only	BioObs	https://doi.org/10.15468/ldch7a	Les Amis de BioObs association	478	478
Citizen science	P-only	Programme national de recensement des observations de requin pèlerin		Association pour l’étude et la conservation des sélaciens (APECS)	1,850	1,850

**Table 2.** Levels of certainty of occurrences.

	Geographically plausible	Geographically implausible
Depth **and** length consistent	Highly probable**	Questionable*
Depth **or** length consistent	Probable**	Questionable*
Depth **or** length inconsistent	Questionable	Questionable
Depth **and** length inconsistent	Invalid	Invalid
* unless expert advice is favourable = Probable		
** unless expert opinion is unfavourable = Questionable		

**Table 3.** List of species occurring in the dataset

Class	Group	Ordre	Family	Species
Holocephali		Chimaeriformes	Chimaeridae	*Chimaera monstrosa* Linnaeus, 1758
				*Chimaera opalescens* Luchetti, Iglésias & Sellos, 2011
				*Hydrolagus affinis* (de Brito Capello, 1868)
				*Hydrolagus mirabilis* (Collett, 1904)
				*Hydrolagus pallidus* Hardy & Stehmann, 1990
			Rhinochimaeridae	*Harriotta raleighana* Goode & Bean, 1895
				*Rhinochimaera atlantica* Holt & Byrne, 1909
Elasmobranchii	Batoidea	Rajiformes	Arhynchobatidae	*Bathyraja pallida* (Forster, 1967)
				*Bathyraja richardsoni* (Garrick, 1961)
			Rajidae	*Amblyraja radiata* (Donovan, 1808)
				*Dipturus batis* (Linnaeus, 1758)
				*Dipturus intermedius* (Parnell, 1837)
				*Dipturus nidarosiensis* (Storm, 1881)
				*Dipturus oxyrinchus* (Linnaeus, 1758)
				*Leucoraja circularis* (Couch, 1838)
				*Leucoraja fullonica* (Linnaeus, 1758)
				*Leucoraja naevus* (Müller & Henle, 1841)
				*Neoraja caerulea* (Stehmann, 1976)
				*Raja asterias* Delaroche, 1809
				*Raja brachyura* Lafont, 1871
				*Raja clavata* Linnaeus, 1758
				*Raja microocellata* Montagu, 1818
				*Raja miraletus* Linnaeus, 1758
				*Raja montagui* Fowler, 1910
				*Raja polystigma* Regan, 1923
				*Raja radula* Delaroche, 1809
				*Raja undulata* Lacepède, 1802
				*Rajella bigelowi* (Stehmann, 1978)
				*Rajella fyllae* (Lütken, 1887)
				*Rajella kukujevi* (Dolganov, 1985)
				*Rostroraja alba* (Lacepède, 1803)
		Myliobatiformes	Dasyatidae	*Bathytoshia lata* (Garman, 1880)
				*Dasyatis pastinaca* (Linnaeus, 1758)
				*Dasyatis tortonesei* Capapé, 1975
				*Pteroplatytrygon violacea* (Bonaparte, 1832)
			Gymnuridae	*Gymnura altavela* (Linnaeus, 1758)
			Mobulidae	*Mobula mobular* (Bonnaterre, 1788)
			Myliobatidae	*Aetomylaeus bovinus* (Geoffroy Saint-Hilaire, 1817)
				*Myliobatis aquila* (Linnaeus, 1758)
		Torpediniformes	Torpedinidae	*Tetronarce nobiliana* (Bonaparte, 1835)
				*Torpedo marmorata* Risso, 1810
				*Torpedo torpedo* (Linnaeus, 1758)
	Selachii	Hexanchiformes	Chlamydoselachidae	*Chlamydoselachus anguineus* Garman, 1884
			Hexanchidae	*Heptranchias perlo* (Bonnaterre, 1788)
				*Hexanchus griseus* (Bonnaterre, 1788)
		Squaliformes	Centrophoridae	*Centrophorus squamosus* (Bonnaterre, 1788)
				*Centrophorus uyato* (Rafinesque, 1810)
				*Deania calceus* (Lowe, 1839)
			Dalatidae	*Dalatias licha* (Bonnaterre, 1788)
			Etmopteridae	*Centroscyllium fabricii* (Reinhardt, 1825)
				*Etmopterus princeps* Collett, 1904
				*Etmopterus pusillus* (Lowe, 1839)
				*Etmopterus spinax* (Linnaeus, 1758)
			Oxynotidae	*Oxynotus centrina* (Linnaeus, 1758)
				*Oxynotus paradoxus* Frade, 1929
			Somniosidae	*Centroscymnus coelolepis* Barbosa du Bocage & de Brito Capello, 1864
				*Centroselachus crepidater* (Barbosa du Bocage & de Brito Capello, 1864)
				*Scymnodon ringens* Barbosa du Bocage & de Brito Capello, 1864
				*Somniosus microcephalus* (Bloch & Schneider, 1801)
				*Somniosus rostratus* (Risso, 1827)
				*Zameus squamulosus* (Günther, 1877)
			Squalidae	*Squalus acanthias* Linnaeus, 1758
				*Squalus blainville* (Risso, 1827)
		Squatinifomes	Squatinidae	*Squatina squatina* (Linnaeus, 1758)
		Orectolobiformes	Ginglymostomatidae	*Ginglymostoma cirratum* (Bonnaterre, 1788)
		Lamniformes	Alopiidae	*Alopias vulpinus* (Bonnaterre, 1788)
			Cetorhinidae	*Cetorhinus maximus* (Gunnerus, 1765)
			Lamnidae	*Carcharodon carcharias* (Linnaeus, 1758)
				*Isurus oxyrinchus* Rafinesque, 1810
				*Lamna nasus* (Bonnaterre, 1788)
		Carcharhiniformes	Carcharhinidae	*Carcharhinus limbatus* (Valenciennes in Müller & Henle, 1839)
				*Carcharhinus longimanus* (Poey, 1861)
				*Carcharhinus plumbeus* (Nardo, 1827)
				*Prionace glauca* (Linnaeus, 1758)
			Pentachiidae	*Apristurus aphyodes* Nakaya & Stehmann, 1998
				*Apristurus melanoasper* Iglésias, Nakaya & Stehmann, 2004
				*Galeus melastomus* Rafinesque, 1810
				*Galeus murinus* (Collett, 1904)
			Pseudotriakidae	*Pseudotriakis microdon* de Brito Capello, 1868
			Scyliorhinidae	*Scyliorhinus canicula* (Linnaeus, 1758)
				*Scyliorhinus stellaris* (Linnaeus, 1758)
			Sphyrnidae	*Sphyrna zygaena* (Linnaeus, 1758)
			Triakidae	*Galeorhinus galeus* (Linnaeus, 1758)
				*Mustelus asterias* Cloquet, 1819
				*Mustelus mustelus* (Linnaeus, 1758)
				*Mustelus punctulatus* Risso, 1827
